# Survey on determinants of intention to reduce nasopharyngeal cancer risk: an application of the theory of planned behavior

**DOI:** 10.1186/s12889-022-14073-0

**Published:** 2022-09-19

**Authors:** Su-Hie Ting, Rayenda Khresna Brahmana, Collin Jerome, Yuwana Podin

**Affiliations:** 1 Faculty of Language and Communication, Universiti Malaysia Sarawak, 94300 Kota Samarahan, Sarawak Malaysia; 2 College of Business Administration, University of Bahrain, Sakhir, 32038 Kingdom of Bahrain; 3 Institute of Health and Community Medicine, Universiti Malaysia Sarawak, 94300 Kota Samarahan, Sarawak Malaysia

**Keywords:** Nasopharyngeal cancer, cancer prevention, Theory of planned behavior, Attitudes, Subjective norm, Perceived behavioral control, Intention

## Abstract

**Background:**

To have better prognostic outcomes and minimize deaths due to nasopharyngeal cancer, it is vital to understand factors that motivate the public to undertake cancer preventive measures. The study investigated determinants of intention to adopt measures to reduce nasopharyngeal cancer risk using the Theory of Planned Behavior.

**Method:**

A cross-sectional survey was conducted on Malaysians (*n* = 515) using a questionnaire on attitudes, subjective norm, perceived behavioral control, knowledge of nasopharyngeal cancer, past nasopharyngeal cancer preventive behavior, and intention to adopt preventive measures. The attitudes construct encompassed perceptions of susceptibility, severity, benefits and barriers. Hierarchical regression of mediation effect under structural equation model approach was used to test the theory. The model was re-estimated using the two-stage least square approach by instrumental approach. Next the Maximum Likelihood Estimation-Structural Equation Modeling was conducted to gauge the instrumentation and check the robustness of the model’s simultaneity.

**Results:**

The respondents had moderate knowledge of nasopharyngeal cancer, and reported high levels of perceived risk, perceived severity and perceived behavioral control. The respondents were under little social pressure (subjective norm) to perform nasopharyngeal cancer preventive actions, marginally believed in the benefits of medical tests and reported few barriers. The Partial Least Squares-Structural Equation Modeling results show that the relationship between intention and four independent variables were significant (perceived behavioral control, perceived risk, perceived severity, marital status) at *p* < .05. Tests of Two-stage Least Square Approach and Maximum Likelihood Estimation-Structural Equation Modeling confirm the four key factors in determining the intention to reduce nasopharyngeal cancer risk. The variance explained by these factors is 33.01 and 32.73% using Two-stage Least Square Approach and Maximum Likelihood Estimation-Structural Equation Modeling respectively. Intention to undertake nasopharyngeal cancer risk-reducing behavior has no significant relationship with subjective norm, attitudes (perceived benefits and barriers to screening), knowledge of nasopharyngeal cancer and past behavior in enacting nasopharyngeal cancer preventive measures. The only demographic variable that affects intention is marital status. Gender, age, race, religion, education level, and income are not significantly associated with intention.

**Conclusions:**

In contexts where knowledge of nasopharyngeal cancer is moderate, the factors associated with the intention to reduce risk are perceived risk and severity, perceived behavioral control, and marital status.

**Supplementary Information:**

The online version contains supplementary material available at 10.1186/s12889-022-14073-0.

## Background

Nasopharyngeal cancer (NPC) is an important public health issue, which is particularly serious for Asian people. NPC is a cancer that develops in the head and neck region. Deaths due to NPC in East and Southeast Asia are particularly high, accounting for 71% of world statistics on NPC mortality. In Malaysia, NPC is now the fourth most common cancer, after breast, colorectal and lung cancers based on The Global Cancer Observatory [1]. Many of the deaths can be avoided if NPC is detected earlier. In Malaysia, patients are usually diagnosed with NPC at stage III or IV (27 and 47%, respectively) [2], leading to poor prognostic outcomes. This is because in the early stages, NPC presents with non-specific symptoms similar to common cold [3]. The early signs are similar to common cold, which is why they are often ignored. NPC may present with nosebleed (which may flow into the throat, causing blood-tinged phlegm), pain or blockage in the ear, loss of hearing, headache, double vision, facial pain, numbness, and a lump in the neck. Balanchandran et al. found that even primary care doctors may not be familiar with uncommon presentations of NPC, causing delayed diagnosis of NPC [2]. In Malaysia, the endeavor to create awareness of NPC currently relies on pamphlets but the public education is not driven by findings on factors that motivate screening uptake to reduce NPC risk.

Little is known about factors that determine motivation to enact NPC preventive measures. Factors determining intention to reduce risk of some cancers have been well studied, particularly cervical cancer [4–11], colorectal cancer [12, 13], and breast cancer [14–16]. Using the Theory of Planned Behavior [17], studies have found that attitude, subjective norm and perceived behavioral control determine intention to undertake cervical cancer screening [4, 6, 11]. Attitude measures an individual’s evaluation or appraisal of the behavior whereas subjective norm measures the effect of perceived social pressure to perform the behavior and perceived behavioral control measures the perceived ease of performing the behavior. The additional determinants of cervical cancer screening intention are the objective promotion factor on advantages and disadvantages of the screening [11], husband’s support behavior [5], knowledge [9], education level [7, 11], and age and childbearing condition [11]. As for colorectal cancer, all three TPB factors predict screening intention [15] but an additional factor is heightened perceived susceptibility in Nigeria [12]. Breast cancer is no different in that all the three TPB factors are predictive of preventive measures like screening [12] and vaccination [18] but the additional factor is perceived susceptibility and benefit [18]. Studies using interviews uncovered other factors affecting breast cancer screening intentions such as language skills and knowledge about breast cancer and screening [14] and communication of genetic risk of breast cancer in the family [16]. The intention to be screened significantly predicted actual cervical cancer screening [8] and Pap smear test [9].

Other findings suggest that certain TPB factors have greater predictive power for certain types of cancer and populations. For oral cancer screening, subjective norm and perceived behavioral control are predictive [19] but for skin cancer, attitude is the main predictor of intention to wear hats, shirt and sunscreen [20]. Attitude and perceived behavioral control predict intention to undertake colorectal cancer screening intention in Hong Kong [13] and HPV screening intention in Canada [7]. As for cervical cancer, increased vaccine intentions are associated with attitude and subjective norm in the United States [21], and with subjective norm and self-efficacy (perceived behavioral control) in Seoul, Korea [22]. Roncancio et al. found that perceived behavioral control is the strongest predictor of Latina’s intention to get a Pap smear test, followed by subjective norm [8]. Thus far, studies using TPB show that attitude is an important determinant of intention to undertake cancer preventive measures, with the exception of some studies [19, 22]. The additional determinants are knowledge (which is associated with education level), perceived susceptibility (which includes family history) and perceived benefits of undertaking the cancer preventive measure. Identification of determinant factors is important in order to target these in public education. Thus far, to our knowledge, studies on determinants of intention to undertake NPC preventive measures have not been conducted.

**Fig. 1:**
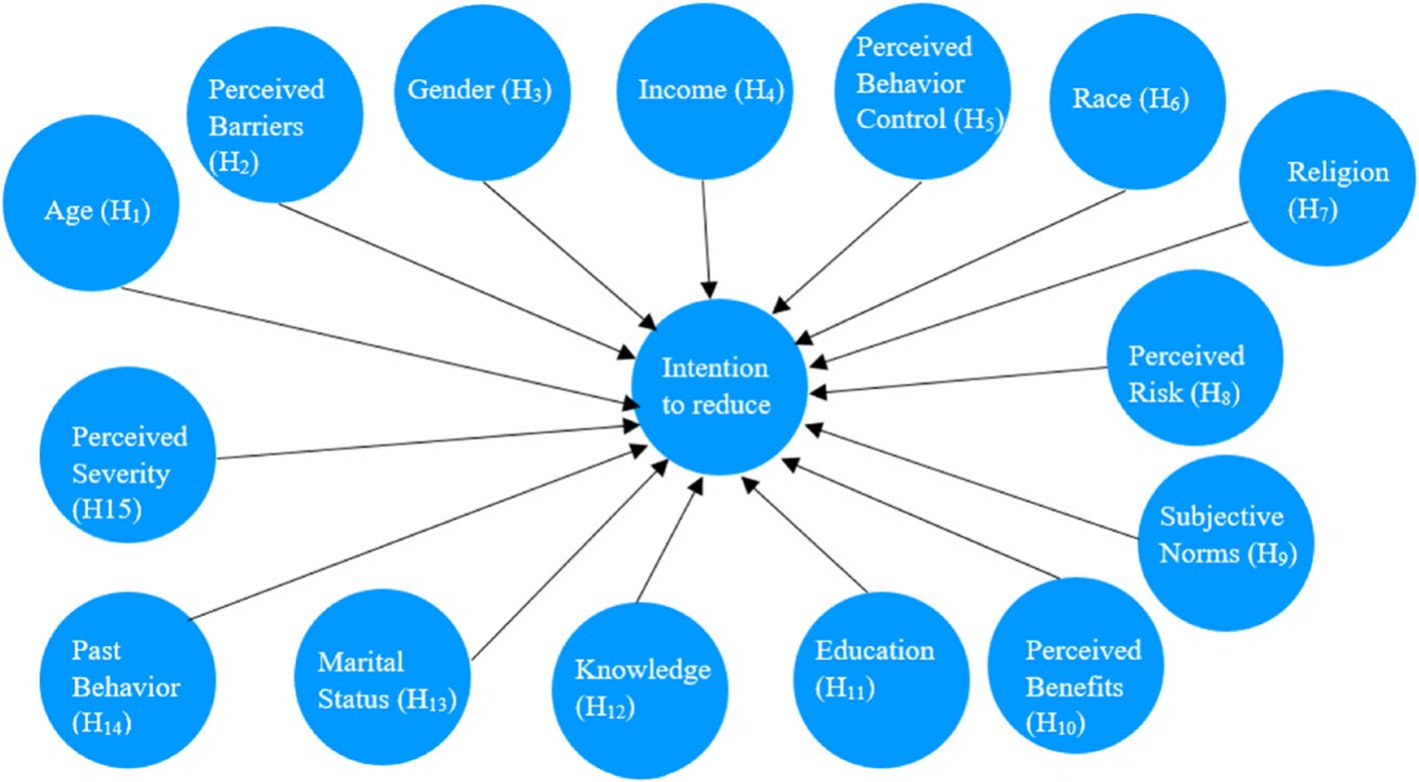
Proposed Model for hypothesis testing

The study investigated determinants of intention to undertake NPC preventive measures in Malaysia. The preventive measures examined were screening and environmental risk factors which are within the volitional control of individuals, that is, reducing consumption of certain preserved food [23], and exposure to environmental pollutants [24, 25]. Figure 1 shows our proposed model for the hypothesis testing.

## Methods

### Respondents

A cross-sectional survey was conducted involving 515 Malaysians aged 15 and above from various ethnic, education and income groups in the states of Penang and Sarawak in Malaysia. The only eligibility criterion for participants was that they had to be Malaysians, regardless of whether or not they had experienced NPC. Following the general rule, the minimum sample size is a five-to-one ratio of the number of independent variables to be tested. However, Hair et al. proposed that the acceptable ratio is ten-to-one [26]. As a list of all the elements of the population was not available, a non-probability sampling or purposive sampling was employed.

Additional file 1 shows the questionnaire used to elicit self-reports of seven independent measures: perceived risk (three items, [27]); perceived severity (eight items, [28, 29]), perceived barriers (10 items, [30]), response efficacy (four items, [31]); self-efficacy (six items, [32]), perceived benefits (4 items, [28]), and subjective norm (four items). The dependent measures consisted of items on intended behavior to reduce NPC risk (four items, [32, 33]). Respondents were also asked to provide health status and demographic information. A seven-point Likert scale was used for each construct of the research model, except for the demographic variables.

Ethical approval for the study was given by the Faculty Medical Ethics Meeting 1/2020 on 4 March 2020 at Universiti Malaysia Sarawak, Malaysia. The ethics committee considered that the investigator had addressed all the issues that might arise in the research and that the investigator had the necessary qualifications, experiences and facilities to conduct the research, and to deal with any emergencies and contingencies that may arise. In addition, the research procedures were in accordance with the Helsinki Declaration of 1975, as revised in 2000 (5).

Respondents were recruited through contacts of researchers and enumerators from workplaces, shopping malls, events, and universities. In addition, we also collected data from villages to avoid the bias of having data from only urban areas. The researchers and enumerators explained the study to respondents who were eligible to participate in the study, and administered the written informed consent and questionnaire face-to-face. Because of this, the response rate was high (98.47%) and only eight turned down the invitation to fill in the questionnaire. Informed consent was obtained from all respondents prior to their participation in the study. For respondents aged 15–18, informed consent from their legal guardians was obtained. The enumerators were trained to answer questions about NPC.

The questionnaires were administered by the researchers and trained enumerators who sat with the participants while they filled in the questionnaire. Thus far, from our fieldwork records, we did not encounter participants who had never heard of NPC. Only one participant in a remote village mistaken NPC for a thyroid swelling and the difference was promptly explained.

### Statistical analysis

We applied robust OLS regression and Partial Least Squares–Structural Equation Model (PLS-SEM) to estimate the determinants of the intention to reduce nasopharyngeal cancer risk by entering the planned behavior factors (subjective norm, perceived behavioral control, perceived risk, perceived severity, perceived benefit, perceived barriers, past behavior, and cancer knowledge) into the model as the contemporaneous variables. The OLS regression serves the baseline estimation of the hypothesis testing. Meanwhile, the PLS-SEM serves a more robust regression estimation. This research also provides a series of robustness check to tackle the theoretical endogeneity issue.

As this research is quantitative by nature, the theory of planned behavior framework is the baseline for the model specification. We added socio-demographic profiles as the control variables to isolate the independent effect of our main effects. The model was estimated using PLS-SEM with the goal of explaining the latent construct variance by minimizing the error terms effect (Hair et al., 2014). Moreover, PLS-SEM imposes less restrictive requirements and assumptions while conserving robustness in estimation (Hair et al., 2014).

The series of goodness of measure tests were run to ensure the reliability and validity of the items and constructs. We describe the results from those tests in the results section. It is noteworthy that that we had run diagnostic tests of classical linear regression model assumptions such as normality, heteroscedasticity, autocorrelation, and multicollinearity after the goodness of measure tests to meet the best linear unbiased estimator’s criteria. Meanwhile, we also ran the post-estimation tests for 2SLS (in our robustness test section) to ensure instrument relevance criterion and exclusion restriction.

## Results

### Characteristics of respondents

Table 1 shows the demographic characteristics of respondents (*n* = 515), with a spread of age, ethnic, educational and income groups that are reflective of the Malaysian population characteristics. The gender distribution is balanced, similar to the Sarawak state (female, 48.23%; male, 51.77%) and Penang (female, 49.72%; male, 50.28%) percentages [34]. The age group information for the Malaysian population is limited to three groups, thereby making a direct comparison difficult (0–14 years old, 25.8%; 15–64 years old, 68.3%; above 65 years old, 5.9%). As for ethnic group, the national distribution is as follows: Malay and indigenous 68.4%, Chinese 23.8% Chinese, 7% Indian, and Others 1%. In the present study, the percentages of Malay and indigenous (combined) and Indian are slightly higher than the national distribution because the study was conducted in Sarawak and Penang respectively, where these two ethnic groups are found in larger numbers. The marital status of the respondents is reflective of the national pattern (single, 34%; married, 55.5%; 3.8% widowed, 1.6% divorced).

**Table 1:** Demographic characteristics of respondents (*n* = 515)

Demographic Variables	%
Gender	
Male	51.57
Female	48.43
Age (Years)	
15–20	16.08
21–30	27.65
31–40	25.69
41–50	11.57
51–60	10.59
> 60	8.42
Ethnic group	
Malay	60.18
Chinese	18.04
Indigenous	15.88
Indian	3.14
Others	2.76
Marital status	
Single	39.03
Married/Divorced/Widowed	60.97
Education	
Primary	2.53
Form 3	5.45
Form 5/Certificate	26.26
Form 6/Diploma/Matriculation	35.99
Bachelor Degree	22.76
Postgraduate Degree	3.89
Professional Qualification	3.12
Income	
No Income	21.55
< RM2,000	20.00
RM2,000-RM3,999	39.03
RM4,000-RM5,999	15.73
> RM6,000	3.69
Knowledge of NPC	
Some knowledge of NPC	52.43
Experienced NPC	5.44
Family experienced NPC	3.69
Work deals with NPC	2.52
Friends and colleagues experienced NPC	18.45
Undertaken medical tests for NPC	2.72
Smoking	
Non-smoker	82.72
Ex-smoker	5.24
Smoker	12.04
Drinking	
Non-drinker	79.03
Occasional drinker	15.53
Moderate drinker	4.47
Heavy drinker	0.97
Consumption of preserved food	
Never	10.68
A few times a year	18.25
Once a month	15.73
Once a week	19.22
A few times a week	36.12
Consumption of salted food	
Never	11.46
A few times a year	20.78
Once a month	23.11
Once a week	17.28
A few times a week	27.37

Information on the extent to which respondents might be at risk of NPC is based on family history and frequency of smoking, drinking, and consumption of preserved food and salted food (Table 1). Only 5.44% of the respondents had NPC and 3.69% had family members who had NPC. Only 12.04% were smokers and 5.44% were moderate to heavy drinkers, but more were at risk due to frequent consumption of preserved food (55.34%) and salted food (44.65%) from once to a few times a week.

### Assessment of goodness of measures

We tested the goodness of measures in the questionnaire to ensure the reliability and validity of the constructs [32]. First, the reliability test, which is a test of how consistently a measuring instrument measures a construct, is assessed by Cronbach’s alpha coefficient. As reported in Table 2, all alpha coefficients are above 0.6, as Nunnaly and Berstein [33] suggested.

**Table 2:** Goodness of measures

Model Construct	Measurement	Item Loading	CR	AVE	Cronbach alpha	Model Construct	Measurement	Item Loading	CR	AVE	Cronbach alpha
Intention	INTENT1	0.879	0.908	0.711	0.864	Perceived benefits	pbenefit1	0.883	0.923	0.750	0.889
	INTENT2	0.843					pbenefit2	0.883			
	INTENT3	0.820					pbenefit3	0.876			
	INTENT4	0.829					pbenefit4	0.821			
Subjective norm	sn1	0.886	0.918	0.736	0.882	Perceived barriers	barrier1	0.809	0.924	0.650	0.922
	sn2	0.895					barrier2	0.823			
	sn3	0.816					barrier3	0.837			
	sn4	0.833					barrier4	0.714			
Perceived behavioral control	pbc1	0.710	0.921	0.662	0.907		barrier5	0.716			
	pbc2	0.719					barrier6	0.744			
	pbc3	0.842					barrier7	0.759			
	pbc4	0.889					barrier8	0.676			
	pbc5	0.862					barrier9	0.650			
	pbc6	0.841					barrier10	0.661			
NPC knowledge	knowledge_1	0.784	0.904	0.610	0.872	Past behavior	past1	0.763	0.861	0.674	0.783
	knowledge_2	0.765					past2	0.849			
	knowledge_3	0.781					past4	0.848			
	knowledge_4	0.764				Perceived risk	prisk1	0.910	0.937	0.832	0.904
	knowledge_5	0.804					prisk2	0.930			
	knowledge_6	0.786					prisk3	0.896			
Perceived severity	severity1	0.779	0.911	0.632	0.883						
	severity4	0.731									
	severity5	0.749									
	severity6	0.836									
	severity7	0.835									
	severity8	0.833									

The validity test, which is a test of how well an instrument that is developed measures the particular concept it is intended to measure, was assessed threefold. First, we tested the construct validity to assess whether or not the measures fit the theories. The items should have 0.5 loadings in their constructs and are not higher than 0.5 across other constructs .

Our observation revealed that all the items fulfill those criteria, thus confirming construct validity. Second, we tested the convergent validity to assess whether or not the items within the same constructs have the same concepts . The requirement for convergent validity is that: (1) loading factors of all items exceeded the recommended value of 0.5, (2) composite reliability has to exceed 0.7, and (3) the Average Variance extracted (AVE) has to exceed 0.5 . Table 2 reports that all values pass the theoretical requirement, implying that our measurements pass the validity test.

Lastly, we tested the discriminant validity, which is a test to reveal the degree to which items differentiate among constructs or measure distinct concepts . To pass the discriminant validity, we have to ensure that items load more strongly on their constructs, and the AVE shared between each construct should be higher than the AVE shared among constructs [35]. Table 3 shows that squared correlations for each construct are less than the average variance extracted by the indicators that indicate adequate discriminant validity.

**Table 3:** Discriminant validity of constructs

	Perceived barriers	Intention	Perceived behavioral control	Perceived risk	Subjective norm	Perceived benefits	Knowledge	Past behavior	Perceived severity
Perceived barriers	0.742								
Intention	−0.251	0.843							
Perceived behavioral control	−0.435	0.285	0.814						
Perceived risk	−0.088	0.441	−0.123	0.912					
Subjective norm	−0.164	0.335	0.107	0.514	0.858				
Perceived benefit	−0.341	0.374	0.456	0.153	0.315	0.866			
Knowledge	−0.273	0.374	0.386	0.236	0.405	0.624	0.781		
Past behavior	−0.441	0.200	0.584	−0.075	0.160	0.391	0.312	0.821	
Perceived severity	−0.361	0.465	0.384	0.295	0.363	0.679	0.609	0.298	0.795

### Regression results

For the hypothesis testing, the results are presented in Table 4. We estimate our proposed model in two regression approaches. First, we run the model under robust OLS regression by clustering the standard errors. The results of the OLS estimation are provided in Column (1). We further examine the causal relationship using the PLS-SEM approach for robustness reasons. While robust OLS regression provides the rigors for the probability of influential observation existence, PLS-SEM provides the factor determinacy by directly estimating latent variable scores for more robust prediction. These two approaches complement each other to retrieve a consistent causal inference. In fact, Table 4 shows our causal inferences are consistent in both models, implying our hypothesis testing is vigorous.

**Table 4:** Regression results

Hypothesis	Relationship	Robust OLS	PLS-SEM	Supported
H_1_	Age and intention	−0.094	− 0.109	No
		(−1.540)	(−1.449)	
H_2_	Barriers and ntention	0.029	−0.011	No
		(0.520)	(0.181)	
H_3_	Gender and intention	−0.124	−0.047	No
		(−1.060)	(−1.061)	
H_4_	Income and intention	0.079	0.068	No
		(0.790)	(0.751)	
H_5_	Perceived behavioral control and intention	0.144^b^	0.211^c^	Yes
		(2.110)	(2.737)	
H_6_	Race and intention	0.036	0.013	No
		(0.330)	(0.087)	
H_7_	Religion and intention	0.047	0.039	No
		(0.370)	(0.25)	
H_8_	Risk and intention	0.240^c^	0.364^c^	Yes
		(3.590)	(4.142)	
H_9_	Subjective norm and intention	0.032	0.016	No
		(0.420)	(0.184)	
H_10_	Benefit and intention	0.048	0.026	No
		(0.560)	(0.334)	
H_11_	Education and intention	−0.004	− 0.012	No
		(− 0.100)	(0.226)	
H_12_	Knowledge and intention	0.039	0.034	No
		(0.450)	(0.485)	
H_13_	Marital status and intention	0.461^c^	0.208^c^	Yes
		(2.760)	(3.115)	
H_14_	Past behavior and intention	0.093	0.069	No
		(1.480)	(1.043)	
H_15_	Severity and intention	0.191^a^	0.144^a^	Yes
		(1.910)	(1.730)	
R2		0.371	0.295	

All reported values are coefficient values except the values inside parentheses, which are T-Values. ^a,b^, and ^c^ denote significance level of 10%, 5%, and 1%, respectively

The results in Table 4 reveal there are three key factors to increase the intention to reduce NPC risk. In the study, the four measures for intention to reduce NPC risk investigated were leading a healthy lifestyle (diet, exercise, avoid smoking), avoiding environmental pollutants, reducing food believed to be linked to NPC (preserved and salted food), and undergoing medical tests for NPC detection (blood test, scanning and biopsy). The intended behavior outcomes examined here are based on the risk factors for NPC [23–25].

First, perceived behavioral control has positive effects on the intention to reduce NPC risk (β = 0.211 *p* < 0.01), implying that an NPC cancer patient with high perceived behavioral control is most likely to have high intention to reduce the risk. This perceived behavioral control is the perceived ability to perform a behavior. For example, if the patients know about the risk of NPC cancer, they are more likely to partake in the activity to reduce the risk. Second, perceived risk also has a positive relationship with the intention to reduce NPC risk (β = 0.364 *p* < 0.01), indicating that an NPC cancer patient with high perceived NPC risk will have a high intention to reduce the risk. This perceived risk refers to the respondents’ subjective judgments about the risk of NPC cancers, such as the illness or mortality incurred from the disease. Given that respondents’ perceived risk is high, it determines their subjective judgment about NPC risk and will increase their intention to reduce it. Finally, the marital status is also a significant factor to increase the intention (β = 0.208 *p* < 0.01), implying that the marital status (married vs. single) is another crucial factor of the respondents’ intention to reduce the NPC risk.

On the other hand, perceived barriers, perceived severity, perceived benefits, knowledge, subjective norms, and past behavior were observed as insignificant factors for reducing NPC risk. For example, the social pressure or the influence from family towards NPC risk (subjective norm) is not a crucial factor for the respondents’ intention to reduce NPC risk. In addition, several demographic variables such as Age, Gender, Income, Race, Religion, and Education also have no significant effect on the respondents’ intention to reduce NPC risk.

**Fig. 2:**
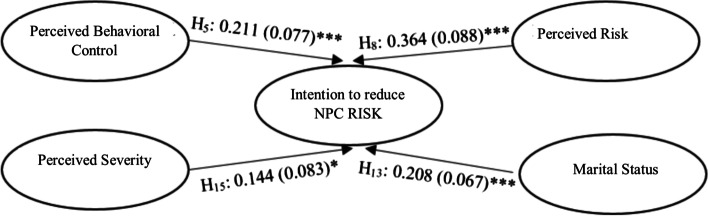
Final estimated model for factors affecting intention to reduce NPC risk

Figure 2 provides a graphical representation of the estimated path coefficient of those accepted hypotheses (full results are provided in Table 4). It surmises that only four hypotheses (out of 15) had a positive relationship with the intention to reduce NPC risk. Therefore, we conclude that perceived behavioral control, perceived risk, marital status, and perceived severity determine the intention to reduce NPC risk in the patients.

### Robustness check: endogeneity test

A robustness check was performed to address the concern of endogeneity, and the results are presented in Table 5. Endogeneity issues will appear due to the concerns for omitted variables that are not included in our models, which is related to a variable we incorporated in our model. It is also due to the simultaneous causality coping where unobserved errors might prevent our study from making causal claims [26, 35, 36]. It is noteworthy we have run the post-estimation diagnostic test to ensure the robustness of the model [37–39]. First, we re-estimated the model using two-stage least square approach (2SLS) by instrumental approach [40]. Given the difficulty of finding a strictly exogenous instrument, we draw upon the previous knowledge and intention studies for our identification strategy. The instrument variables follow the literature of knowledge and intention [41–43], whereas the determinants of knowledge-intention are self-efficacy, experience, and supportive environment. Those three determinants are our instrumental variables for the first stage estimation of our 2SLS model. The results for this cross-sectional 2SLS reveal that our main conclusion on the positive impact of perceived behavioral control, perceived risk, and perceived severity remain intact. Second, we followed Barroso et al. [44] and Asosega et al., [45] to conduct Maximum Likelihood Estimation-Structural Equation Modeling (MLE-SEM), where it gauges the instrumentation and ensures the robustness of the model’s simultaneity. MLE-SEM is a causal modeling approach aimed at maximizing the explained variance of the dependent latent constructs based on its maximum-likelihood estimation. It allows the estimation for a system of equations, where the variables (the constructs) may be measured with error, and this error may have interrelationship with other errors from the same constructs or from other constructs. This technique employs an iterative procedure to minimize the discrepancy between the sample covariance matrix and the reproduced covariance matrix, evaluated by a fit function. Further, this method generalizes iterative estimation of latent constructs and shows the interrelationships (paths) between latent constructs [44–46]. Hence, the structural equation model only permits the structural path between the latent constructs in a single direction. Note that we ran diagnostic test to ensure our estimation meets the classical linear regression assumptions, yet, our pre-estimations showed that our estimation models have passed the diagnostic test (reject null hypothesis).

**Table 5:** Endogeneity test results

	2SLS		MLE-SEM	
	coefficient	standard errors	coefficient	standard errors
Knowledge	0.003	0.085	0.039	0.079
Subjective norm	0.038	0.054	0.032	0.054
Perceived behavioral control	0.149^**^	0.061	0.144^**^	0.061
Perceived risk	0.240^***^	0.041	0.240^***^	0.041
Perceived severity	0.199^**^	0.087	0.191^**^	0.087
Benefit	0.059	0.078	0.048	0.078
Barrier	0.031	0.045	0.029	0.045
Past behavior	0.092	0.062	0.093	0.062
Gender	−0.119	0.120	−0.124	0.120
Age	−0.090	0.058	−0.094	0.058
Race	0.038	0.200	0.036	0.200
Religion	0.045	0.200	0.047	0.200
Marital status	0.459^***^	0.149	0.461^***^	0.149
Education	−0.006	0.038	−0.004	0.038
Income	0.081	0.074	0.079	0.074
Constant	0.875	0.569	0.851	0.569

*p < .05, **p < .01, **p < .001

The results from this approach have the same conclusion as our earlier results, reaffirming that perceived behavioral control, perceived risk, perceived severity and marital status are key factors in determining the intention to reduce NPC risk. The variance explained by these factors using PLS-SEM is 29.5% (Table 4), 33.01% and 32.73% using 2SLS and MLE-SEM respectively (Table 5).

## Discussion

The present study on the viability of TPB to predict intention to adopt NPC preventive measures in a sample of Malaysians produced two noteworthy findings.

Firstly, two TPB components, attitude (perceived risk, perceived severity) and perceived behavioral control, have significant relationship with intention. The two sub-constructs of attitude (risk and severity) that determine NPC risk-reducing intention constitutes threat posed by the cancer. In the case of NPC for Malaysians, the risk is high because NPC incidence ranks fourth among the cancers, and the respondents were worried about getting or inheriting the cancer. The respondents perceived NPC as bringing severe consequences such as inability to speak, physical deformity, severe pain, and death. The high levels of perceived risk and perceived severity construct NPC as a threatening disease, and respondents who believed that they could perform NPC risk-reducing behaviors (high perceived behavioral control) reported greater intention to take NPC preventive measures such as looking for information on NPC, leading a healthy lifestyle (diet, exercise, avoid smoking), avoiding environmental pollutants, reducing food believed to be associated with NPC (preserved and salted food), and undergoing medical tests for NPC detection. Based on qualitative analysis of interviews, researchers have found that intention to have breast cancer screening is higher with awareness of heightened risk due to family history [16] and more knowledge of the disease [14]. Wang et al. also found that perceived high susceptibility and beliefs on cervical cancer as a behavior-preventable disease accounted for 47% of variance in parents’ intention to vaccinate their daughters against HPV [10]. Perceived behavioral control having a significant effect on intention is not surprising in view of similar findings on oral cancer [19], colorectal cancer screening [13], HPV screening [7], and pap smear test [8].

The results of the present study showed that intention to undertake NPC risk-reducing behavior has no relationship with subjective norm, attitude (perceptions of benefits and barriers) and knowledge of NPC. Interestingly, studies have found that all the three TPB elements determine intention to undertake preventive measures for other cancers like cervical cancer [4, 6, 11], colorectal cancer [12, 15], and breast cancer [15, 18]. Subjective norm was found to be predictive of intention for oral cancer screening [19] and cervical cancer vaccination [8, 21, 22]. The different results of the present study on NPC will be discussed to understand the implications on public health concerns.

On subjective norm, the findings indicate that there was little social pressure on the respondents to perform NPC preventive actions (leading a healthy lifestyle, avoiding environmental pollutants, reducing food believed to be associated with NPC, and undergoing medical tests for NPC detection such as physical examination, blood test, scan, biopsy). Most of the people close to the respondents may lack awareness of the threat posed by NPC, thereby explaining the lack of association between subjective norm and intention to undertake NPC risk-reducing behaviors.

Out of the four NPC preventive measures, we focused on medical testing for further investigation on the respondents’ attitudes because the other three NPC preventive actions were less definitive. The sub-constructs of attitudes investigated were perceptions of benefits and barriers of undertaking medical testing, and the results showed non-significance of association with intention. The respondents marginally believed in the benefits of medical tests for NPC detection, and did not have much barriers such as lack of information, fear, embarrassment, time, pain, transportation problems and distrust of the medical results. These results suggest that in health risk communication on NPC in contexts similar to the present study, it is not necessary to clutter the messages with information addressing benefits and barriers of health protective measures.

The respondents had moderate knowledge of NPC. In the present study, knowledge of NPC was measured based on direct and indirect experiences with NPC, including the respondent or close contacts (family, friends) having the cancer, work dealing with NPC and having undergone medical tests for NPC. Besides knowledge, past behavior in enacting NPC preventive measures also did not determine intention. Other studies have measured knowledge using tests of facts for diseases [9, 47, 48]. For example, Sarvestani et al. measured knowledge of cervical cancer using 15 yes/no questions, and found knowledge to be positively associated with screening intention [9]. 

In the interest of public health, the results on determinants of intention to undertake NPC risk-reducing behaviors indicate that health communication on NPC should include information on risk and severity of NPC, as well as motivational messages to heighten perceived behavioral control and self-efficacy in adopting NPC preventive measures. It is important to build the confidence of the public in their ability to take preventive measures. Studies have found that individuals who hold cancer fatalistic beliefs are less likely to seek information on cancer [49] and engage in screening behaviors [50]. Health risk communication needs to target cancer fatalism and pessimistic beliefs about the impossibility of preventing and treating cancer. This is because survival rates for people diagnosed with NPC in the early states are encouraging, and self-efficacy is needed for them to adopt health protective measures.

The second finding on marital status being the only demographic variable that affects intention is fresh. Respondents who are married reported greater intention to undertake NPC risk-reducing behaviors. This finding is novel because other studies have identified education level [7, 11], income and age [11] as the determinants for screening intention. This finding is reflective of sociocultural context having a significant relationship with cancer risk perceptions. It is probable that married individuals are worried about the dire consequences on their family and dependents should they contract NPC. It is also probable that they have a wider social circle because when they are in two families, they are in contact with more news on people suffering from NPC, and this could have created more awareness of the need for regular screening.

## Conclusions

This is the first study to use the TPB framework to understand determinants of intention to undertake NPC risk-reducing behaviors in a context of high NPC incidence and moderate knowledge of NPC. The study showed that attitude (perceived risk, perceived severity) and perceived behavioral control determine intention. The only demographic variable that affects intention is marital status and this is a new finding, in comparison to other studies which have identified education level [7, 11], income and age [11]. The results suggest that further research needs to be conducted on the message framing of NPC concerns for single and married individuals to find out how it affects their knowledge of NPC and intention to undertake preventive measures. A limitation of the present study is that NPC knowledge was assessed only through self-reports on whether they had heard of NPC, their own experience of NPC and that of their family and friends, and whether their work was related to NPC and if they had undergone NPC screening. While experiential knowledge of NPC is desirable, an additional measure in the form of tests of facts [9, 48, 49] on NPC can strengthen the measure of NPC knowledge. Whether or not knowledge tests on NPC are better in predicting intention than direct/indirect experiential knowledge needs to be investigated in future research. Another limitation of the study is the non-random sampling, which carries potential implications for selection biases and limits generalisation of the findings to other populations. Further studies dealing with the effectiveness of educational interventions involving various message designs will lead to a better understanding of health behavior change and the development of effective health education materials. Econometrically, our methods have several limitations. For instance, our identification strategy for 2SLS is straightforward due to the difficulty of finding a strictly exogeneous external instruments. There is possibility that other exogeneous external instruments are more fit to build the estimation. Meanwhile, the MLE-SEM focuses more on empirical covariance of all indicator variables which are more suitable as a confirmatory approach. However, our research does not serve the debate about the econometrical issue, we open the discussion for future research.

## Supplementary Information

**Additional file 1.**

## Data Availability

The datasets for the study are available from the corresponding author upon reasonable request.
