# Supplementary material for: Survey on determinants of intention to reduce nasopharyngeal cancer risk: an application of the theory of planned behavior
Source: BMC Public Health. 2022 Sep 19;22:1774. doi: 10.1186/s12889-022-14073-0 (PMC9487021; doi:10.1186/s12889-022-14073-0)
Supplement: Supplementary file 1 — Additional file 1. [file 12889_2022_14073_MOESM1_ESM.doc]

**Additional file 1: QUESTIONNAIRE** ON PERCEPTIONS TOWARDS NOSE AND THROAT CANCER IN MALAYSIA

| **Section I – Demographic Background** |
| --- |

**Instruction: Please indicate your response by marking ✔ in the most appropriate box.**

| **1.** | **Gender** | ⬜ Male | | | | | | ⬜ Female | | | | | | |
| --- | --- | --- | --- | --- | --- | --- | --- | --- | --- | --- | --- | --- | --- | --- |
| **2.** | **Age** | ⬜ ≤20 | ⬜ 21-30 | | | ⬜ 31-40 | | ⬜ 41-50 | | ⬜ 51-60 | | | ⬜ ≥ 61 | |
| **3.** | **Race** | ⬜ Malay  ⬜ Chinese, please state your dialect group ……………………  ⬜ Sarawak Indigenous, please state your ethnic group …………………………  ⬜ Indian  ⬜ Others, please specify …………  ⬜ Mixed, please specify ………… | | | | | | | | | | | | |
| **4.** | **Religion** | ⬜ Islam | | ⬜ Christian | | | ⬜ Buddhist | | ⬜ Hindu | | ⬜ Others | | |  |
| **5.** | **Marital Status** | ⬜ Single | | | ⬜ Married | | | | ⬜ Divorced | | | ⬜ Widowed | | |
| **6.** | **Occupation** | ⬜ Not working  ⬜ Professional / Technical  ⬜ Manager / Official / Proprietor  ⬜ Salesman  ⬜ Business Owner / Operator  ⬜ Public Servant  ⬜ Labourer  ⬜ Service Worker | | | | | | | ⬜ Disability  ⬜ Student  ⬜ Retired  ⬜ Others, please specify  …………………. | | | | | |
| **7.** | **Field of Occupation** | ⬜ Accounting  ⬜ Agriculture  ⬜ Banking  ⬜ Business  ⬜ Construction  ⬜ Distribution-Shipping  ⬜ Education  ⬜ Engineering  ⬜ Grocery  ⬜ Health Care | | | | | | | ⬜ Hotel  ⬜ Insurance  ⬜ Manufacturing/Production  ⬜ Marketing  ⬜ Media  ⬜ Restaurant  ⬜ Telecommunications  ⬜ Transportation  ⬜ Others, please specify  ………………….……… | | | | | |
| **8.** | **Highest Educational Attainment** | ⬜ No formal schooling  ⬜ Primary 6 or below  ⬜ Form 3  ⬜ Form 5  ⬜ Certificate  ⬜ Form 6/Diploma/Matriculation | | | | | | | ⬜ Bachelor’s Degree  ⬜ Master’s Degree  ⬜ Doctorate Degree  ⬜ Professional Qualification  ⬜ Others, please specify  ………………………………. | | | | | |
| **9.** | **Monthly salary** | ⬜ Not working  ⬜ Less than RM2,000  ⬜ RM2,000-RM3,999  ⬜ RM4,000-RM5,999  ⬜ RM6,000-RM7,999  ⬜ RM8,000-RM9,999  ⬜ More than RM10,000 | | | | | | | | | | | | |
| **10.** | **Smoking Status** | ⬜ Non-Smoker  ⬜ Ex-Smoker, stopped for how long: …………………………….  ⬜ Current Smoker, and have been smoking for how long: …………………….. | | | | | | | | | | | | |
| **11.** | **Drinking Behaviour** | ⬜ Non-Drinker  ⬜ Occasional Social Drinker  ⬜ Moderate Drinker  ⬜ Heavy Drinker | | | | | | | | | | | | |
| **12.** | **How often do you eat preserved food** (*e.g., vegetables and meat*)? | | | | | | | | ⬜ Never  ⬜ A few times a year  ⬜ At least once a month  ⬜ At least once a week  ⬜ A few times a week | | | | | |
| **13.** | **How often do you eat salted food** (*e.g., salted eggs, salted fish*)**?** | | | | | | | | ⬜ Never  ⬜ A few times a year  ⬜ At least once a month  ⬜ At least once a week  ⬜ A few times a week | | | | | |

| **Section 2 – Knowledge of Nose and Throat Cancer** |
| --- |

**Instruction: Please indicate your response by marking ✔ in the most appropriate box.**

| **14.** | Do you **know anything** about nose and throat cancer? | ⬜ No  ⬜ Yes |
| --- | --- | --- |
| **15.** | Have you **ever had** nose and throat cancer before this? | ⬜ No  ⬜ Yes |
| **16.** | Does your **work deal** with nose and throat cancer? | ⬜ No  ⬜ Yes |
| **17.** | Do you have **family who have** nose and throat cancer? | ⬜ No  ⬜ Yes, who? …………………………….…….. |
| **18.** | Do you know of **friends and colleagues who have** nose and throat cancer? | ⬜ No  ⬜ Yes |
| **19.** | Have you **done medical tests** for nose and throat cancer? | ⬜ No  ⬜ Yes |

| **Section 3 – Perceived Risk of Getting Nose and Throat Cancer** |
| --- |

**Instruction: Kindly indicate your response by marking (✔) in the most appropriate box.**

|  | **20.** | | Nose and throat cancer **could happen to me**. | | | | | | |  | |  |
| --- | --- | --- | --- | --- | --- | --- | --- | --- | --- | --- | --- | --- |
|  |  | ⬜ 1 Strongly disagree | | ⬜ 2 Disagree | | ⬜ 3 Somewhat disagree | ⬜ 4 Neutral | ⬜ 5 Somewhat agree | ⬜ 6 Agree | | ⬜ 7 Strongly agree |  |
|  | **21.** | | How **likely are you to get** nose and throat cancer? | | | | | | | | |  |
|  |  | | ⬜ 1 Very low | | ⬜ 2 Low | ⬜ 3 Somewhat low | ⬜ 4 Medium | ⬜ 5 Somewhat high | ⬜ 6 High | | ⬜ 7 Very high |  |
|  | **22.** | | How likely are you to get nose and throat cancer **compared with other people your age**? | | | | | | | | |  |
|  |  | | ⬜ 1 very much lower than others | | ⬜ 2 lower than others | ⬜ 3 a little lower than others | ⬜ 4 equal to others | ⬜ 5 a little higher than others | ⬜ 6 higher than others | | ⬜ 7 very much higher than others |  |
| **Section 4 – Perceived Severity of Nose and Throat Cancer** | | | | | | | | | | | | |

**Instruction: Kindly indicate your response by marking (✔) in the most appropriate box.**

| **23.** | I am **worried** **about getting nose and throat cancer** in my lifetime. | | | | | | |
| --- | --- | --- | --- | --- | --- | --- | --- |
|  | ⬜ 1 Strongly disagree | ⬜ 2 Disagree | ⬜ 3 Somewhat disagree | ⬜ 4 Neutral | ⬜ 5 Somewhat agree | ⬜ 6 Agree | ⬜ 7 Strongly agree |
| **24.** | I am **worried about inheriting** nose and throat cancer from my family. | | | | | | |
|  | ⬜ 1 Strongly disagree | ⬜ 2 Disagree | ⬜ 3 Somewhat disagree | ⬜ 4 Neutral | ⬜ 5 Somewhat agree | ⬜ 6 Agree | ⬜ 7 Strongly agree |
| **25.** | If I get nose and throat cancer, I would **feel imperfect**. | | | | | | |
|  | ⬜ 1 Strongly disagree | ⬜ 2 Disagree | ⬜ 3 Somewhat disagree | ⬜ 4 Neutral | ⬜ 5 Somewhat agree | ⬜ 6 Agree | ⬜ 7 Strongly agree |
| **26.** | I am **afraid to even think** about nose and throat cancer. | | | | | | |
|  | ⬜ 1 Strongly disagree | ⬜ 2 Disagree | ⬜ 3 Somewhat disagree | ⬜ 4 Neutral | ⬜ 5 Somewhat agree | ⬜ 6 Agree | ⬜ 7 Strongly agree |
| **27.** | Nose and throat cancer **can kill**. | | | | | | |
|  | ⬜ 1 Strongly disagree | ⬜ 2 Disagree | ⬜ 3 Somewhat disagree | ⬜ 4 Neutral | ⬜ 5 Somewhat agree | ⬜ 6 Agree | ⬜ 7 Strongly agree |
| **28.** | Nose and throat cancer can cause **a lot of pain**. | | | | | | |
|  | ⬜ 1 Strongly disagree | ⬜ 2 Disagree | ⬜ 3 Somewhat disagree | ⬜ 4 Neutral | ⬜ 5 Somewhat agree | ⬜ 6 Agree | ⬜ 7 Strongly agree |
| **29.** | Nose and throat cancer can make the person **unable to speak**. | | | | | | |
|  | ⬜ 1 Strongly disagree | ⬜ 2 Disagree | ⬜ 3 Somewhat disagree | ⬜ 4 Neutral | ⬜ 5 Somewhat agree | ⬜ 6 Agree | ⬜ 7 Strongly agree |
| **30.** | Nose and throat cancer can cause the person to **have physical deformity**. | | | | | | |
|  | ⬜ 1 Strongly disagree | ⬜ 2 Disagree | ⬜ 3 Somewhat disagree | ⬜ 4 Neutral | ⬜ 5 Somewhat agree | ⬜ 6 Agree | ⬜ 7 Strongly agree |

| **Section 5 – Perceived Benefits of Medical Tests for Nose and Throat Cancer** |
| --- |

**Instruction: Kindly indicate your response by marking (✔) in the most appropriate box.**

| **31.** | | When I go for medical tests (*e.g., physical examination, blood test, scan, biopsy*) to detect nose and throat cancer, **I am** **doing something to take care of myself**. | | | | | | | | | | | | |
| --- | --- | --- | --- | --- | --- | --- | --- | --- | --- | --- | --- | --- | --- | --- |
|  | | ⬜ 1 Strongly disagree | | ⬜ 2 Disagree | | ⬜ 3 Somewhat disagree | | ⬜ 4 Neutral | | ⬜ 5 Somewhat agree | | ⬜ 6 Agree | | ⬜ 7 Strongly agree |
| **32.** | | When I go for medical tests, it is because **I want to know whether I have** nose and throat cancer. | | | | | | | | | | | | |
|  | | ⬜ 1 Strongly disagree | | ⬜ 2 Disagree | | ⬜ 3 Somewhat disagree | | ⬜ 4 Neutral | | ⬜ 5 Somewhat agree | | ⬜ 6 Agree | | ⬜ 7 Strongly agree |
| **33.** | | If I find signs of nose and throat cancer through medical tests, **I can get early treatment**. | | | | | | | | | | | | |
|  | | ⬜ 1 Strongly disagree | | ⬜ 2 Disagree | | ⬜ 3 Somewhat disagree | | ⬜ 4 Neutral | | ⬜ 5 Somewhat agree | | ⬜ 6 Agree | | ⬜ 7 Strongly agree |
| **34.** | If I find signs of nose and throat cancer earlier through medical tests, **my chance of recovery is higher**. | | | | | | | | | | | | | |
|  | ⬜ 1 Strongly disagree | | ⬜ 2 Disagree | | ⬜ 3 Somewhat disagree | | ⬜ 4 Neutral | | ⬜ 5 Somewhat agree | | ⬜ 6 Agree | | ⬜ 7 Strongly agree | |

| **Section 6 – Perceived Barriers of Going for Medical Tests for Nose and Throat Cancer** |
| --- |

**Instruction: Kindly indicate your response by marking (✔) in the most appropriate box.**

|  | **35.** | | I have **no idea how to go about getting medical tests** for nose and throat cancer (*e.g., physical examination, blood test, scan, biopsy*). | | | | | | |  |
| --- | --- | --- | --- | --- | --- | --- | --- | --- | --- | --- |
|  |  | ⬜ 1 Strongly disagree | | ⬜ 2 Disagree | ⬜ 3 Somewhat disagree | ⬜ 4 Neutral | ⬜ 5 Somewhat agree | ⬜ 6 Agree | ⬜ 7 Strongly agree |  |
|  | **36.** | | I would not go for medical tests for nose and throat cancer because **I am afraid to know whether I have it**. | | | | | | |  |
|  |  | ⬜ 1 Strongly disagree | | ⬜ 2 Disagree | ⬜ 3 Somewhat disagree | ⬜ 4 Neutral | ⬜ 5 Somewhat agree | ⬜ 6 Agree | ⬜ 7 Strongly agree |  |
|  | **37.** | | I am afraid to go for medical tests for nose and throat cancer because I **do not know what to do with the test results**. | | | | | | |  |
|  |  | ⬜ 1 Strongly disagree | | ⬜ 2 Disagree | ⬜ 3 Somewhat disagree | ⬜ 4 Neutral | ⬜ 5 Somewhat agree | ⬜ 6 Agree | ⬜ 7 Strongly agree |  |
|  | **38.** | | I would not go for medical tests for nose and throat cancer because it is **embarrassing**. | | | | | | |  |
|  |  | ⬜ 1 Strongly disagree | | ⬜ 2 Disagree | ⬜ 3 Somewhat disagree | ⬜ 4 Neutral | ⬜ 5 Somewhat agree | ⬜ 6 Agree | ⬜ 7 Strongly agree |  |
|  | **39.** | | I would not go for medical tests for nose and throat cancer because it would **take too much time**. | | | | | | |  |
|  |  | ⬜ 1 Strongly disagree | | ⬜ 2 Disagree | ⬜ 3 Somewhat disagree | ⬜ 4 Neutral | ⬜ 5 Somewhat agree | ⬜ 6 Agree | ⬜ 7 Strongly agree |  |
|  | **40.** | | I would not go for medical tests for nose and throat cancer because it would be **painful**. | | | | | | |  |
|  |  | ⬜ 1 Strongly disagree | | ⬜ 2 Disagree | ⬜ 3 Somewhat disagree | ⬜ 4 Neutral | ⬜ 5 Somewhat agree | ⬜ 6 Agree | ⬜ 7 Strongly agree |  |
|  | **41.** | | I would not go for medical tests for nose and throat cancer because I **distrust the test results.** | | | | | | |  |
|  |  | ⬜ 1 Strongly disagree | | ⬜ 2 Disagree | ⬜ 3 Somewhat disagree | ⬜ 4 Neutral | ⬜ 5 Somewhat agree | ⬜ 6 Agree | ⬜ 7 Strongly agree |  |
|  | **42.** | | It is **difficult to get transportation** to go for medical tests for nose and throat cancer. | | | | | | |  |
|  |  | ⬜ 1 Strongly disagree | | ⬜ 2 Disagree | ⬜ 3 Somewhat disagree | ⬜ 4 Neutral | ⬜ 5 Somewhat agree | ⬜ 6 Agree | ⬜ 7 Strongly agree |  |
|  | **43.** | | I have **other problems more important** than getting medical tests for nose and throat cancer. | | | | | | |  |
|  |  | ⬜ 1 Strongly disagree | | ⬜ 2 Disagree | ⬜ 3 Somewhat disagree | ⬜ 4 Neutral | ⬜ 5 Somewhat agree | ⬜ 6 Agree | ⬜ 7 Strongly agree |  |
|  | **44.** | | Medical tests for nose and throat cancer **cost too much money**. | | | | | | |  |
|  |  | ⬜ 1 Strongly disagree | | ⬜ 2 Disagree | ⬜ 3 Somewhat disagree | ⬜ 4 Neutral | ⬜ 5 Somewhat agree | ⬜ 6 Agree | ⬜ 7 Strongly agree |  |
| **Section 7 – Response Efficacy: Confidence in Effectiveness of Preventive Measures** | | | | | | | | | | |

**Instruction: Kindly indicate your response by marking (✔) in the most appropriate box.**

|  | **45.** | How confident are you that **medical tests** (*e.g., physical examination, blood test, scan, biopsy*) can reduce the risk of nose and throat cancer? | | | | | | |  |
| --- | --- | --- | --- | --- | --- | --- | --- | --- | --- |
|  |  | ⬜ 1 Very not confident | ⬜ 2 Not confident | ⬜ 3 Somewhat not confident | ⬜ 4 Neutral | ⬜ 5 Somewhat confident | ⬜ 6 Confident | ⬜ 7 Very confident |  |
|  | **46.** | How confident are you that **changing to a healthier lifestyle** (e.g., *diet, exercise*) can reduce the risk of nose and throat cancer? | | | | | | |  |
|  |  | ⬜ 1 Very not confident | ⬜ 2 Not confident | ⬜ 3 Somewhat not confident | ⬜ 4 Neutral | ⬜ 5 Somewhat confident | ⬜ 6 Confident | ⬜ 7 Very confident |  |
|  | **47.** | How confident are you that **avoiding certain food** (e.g., *preserved vegetables/meat, salted eggs/fish*) can reduce the risk of nose and throat cancer? | | | | | | |  |
|  |  | ⬜ 1 Very not confident | ⬜ 2 Not confident | ⬜ 3 Somewhat not confident | ⬜ 4 Neutral | ⬜ 5 Somewhat confident | ⬜ 6 Confident | ⬜ 7 Very confident |  |
|  | **48.** | How confident are you that **avoiding environmental pollutants** can reduce the risk of nose and throat cancer? | | | | | | |  |
|  |  | ⬜ 1 Very not confident | ⬜ 2 Not confident | ⬜ 3 Somewhat not confident | ⬜ 4 Neutral | ⬜ 5 Somewhat confident | ⬜ 6 Confident | ⬜ 7 Very confident |  |
| **Section 8 – Self-Efficacy: Confidence to Take Action to Reduce Risk to Nose and Throat Cancer** | | | | | | | | | |

**Instruction: Kindly indicate your response by marking (✔) in the most appropriate box.**

| **49.** | How **confident** are you that you **can change to a healthier lifestyle** (e.g., *diet, exercise, avoid smoking*) to reduce risk to nose and throat cancer? | | | | | | |
| --- | --- | --- | --- | --- | --- | --- | --- |
|  | ⬜ 1 Very not confident | ⬜ 2 Not confident | ⬜ 3 Somewhat not confident | ⬜ 4 Neutral | ⬜ 5 Somewhat confident | ⬜ 6 Confident | ⬜ 7 Very confident |
| **50.** | How **easy** is it for you to **change to a healthier lifestyle** to reduce risk to nose and throat cancer? | | | | | | |
|  | ⬜ 1 Very difficult | ⬜ 2 Difficult | ⬜ 3 Somewhat difficult | ⬜ 4 Neutral | ⬜ 5 Somewhat easy | ⬜ 6 Easy | ⬜ 7 Very easy |
| **51.** | How **confident** are you that you **can** **avoid certain food** said to cause nose and throat cancer to reduce risk to the disease? | | | | | | |
|  | ⬜ 1 Very not confident | ⬜ 2 Not confident | ⬜ 3 Somewhat not confident | ⬜ 4 Neutral | ⬜ 5 Somewhat confident | ⬜ 6 Confident | ⬜ 7 Very confident |
| **52.** | How **easy** is it for you to **avoid certain food** believed to cause nose and throat cancer to reduce risk to the disease? | | | | | | |
|  | ⬜ 1 Very difficult | ⬜ 2 Difficult | ⬜ 3 Somewhat difficult | ⬜ 4 Neutral | ⬜ 5 Somewhat easy | ⬜ 6 Easy | ⬜ 7 Very easy |
| **53.** | How **confident** are you that you **can go for medical tests** for nose and throat cancer for early detection/treatment of nose and throat cancer? | | | | | | |
|  | ⬜ 1 Very not confident | ⬜ 2 Not confident | ⬜ 3 Somewhat not confident | ⬜ 4 Neutral | ⬜ 5 Somewhat confident | ⬜ 6 Confident | ⬜ 7 Very confident |
| **54.** | How **easy** is it for you to **go for medical tests** for nose and throat cancer for early detection/treatment of nose and throat cancer? | | | | | | |
|  | ⬜ 1 Very difficult | ⬜ 2 Difficult | ⬜ 3 Somewhat difficult | ⬜ 4 Neutral | ⬜ 5 Somewhat easy | ⬜ 6 Easy | ⬜ 7 Very easy |

| **Section 9 – Subjective Norm** |
| --- |

**Instruction: Kindly indicate your response by marking (✔) in the most appropriate box.**

| **55.** | | Most people who are important to me think I should **go for medical tests** for nose and throat cancer (*e.g., physical examination, blood test, scan, biopsy*). | | | | | | |
| --- | --- | --- | --- | --- | --- | --- | --- | --- |
|  | ⬜ 1 Strongly disagree | | ⬜ 2 Disagree | ⬜ 3 Somewhat disagree | ⬜ 4 Neutral | ⬜ 5 Somewhat agree | ⬜ 6 Agree | ⬜ 7 Strongly agree |
| **56.** | | Most people who are important to me think I should **change to a healthier lifestyle** (e.g., *diet, exercise, avoid smoking*) to reduce risk of getting nose and throat cancer. | | | | | | |
|  | ⬜ 1 Strongly disagree | | ⬜ 2 Disagree | ⬜ 3 Somewhat disagree | ⬜ 4 Neutral | ⬜ 5 Somewhat agree | ⬜ 6 Agree | ⬜ 7 Strongly agree |
| **57.** | | Most people who are important to me think I should **avoid food believed to cause nose and throat cancer** (*e.g., preserved vegetables/meat, salted eggs/fish*). | | | | | | |
|  | ⬜ 1 Strongly disagree | | ⬜ 2 Disagree | ⬜ 3 Somewhat disagree | ⬜ 4 Neutral | ⬜ 5 Somewhat agree | ⬜ 6 Agree | ⬜ 7 Strongly agree |
| **58.** | | Most people who are important to me think I should do something (*e.g., change job, move to another place*) to **avoid environmental** **pollutants** which may cause nose and throat cancer. | | | | | | |
|  | ⬜ 1 Strongly disagree | | ⬜ 2 Disagree | ⬜ 3 Somewhat disagree | ⬜ 4 Neutral | ⬜ 5 Somewhat agree | ⬜ 6 Agree | ⬜ 7 Strongly agree |

| **Section 10 – Intended Behavior** |
| --- |

**Instruction: Kindly indicate your response by marking (✔) in the most appropriate box.**

| **59.** | | | I intend to lead a healthier lifestyle after today  **to** **reduce the risk of getting nose and throat cancer**. | | | | | | |
| --- | --- | --- | --- | --- | --- | --- | --- | --- | --- |
|  | ⬜ 1 Strongly disagree | | | ⬜ 2 Disagree | ⬜ 3 Somewhat disagree | ⬜ 4 Neutral | ⬜ 5 Somewhat agree | ⬜ 6 Agree | ⬜ 7 Strongly agree |
| **60.** | | I intend to avoid environmental pollutants **after today to reduce the risk of getting nose and throat cancer**. | | | | | | | |
|  | ⬜ 1 Strongly disagree | | | ⬜ 2 Disagree | ⬜ 3 Somewhat disagree | ⬜ 4 Neutral | ⬜ 5 Somewhat agree | ⬜ 6 Agree | ⬜ 7 Strongly agree |
| **61.** | | I intend to reduce food believed to cause nose and throat cancer (*e.g., preserved vegetables/meat, salted eggs/fish*) **to reduce the risk of getting nose and throat cancer after today**. | | | | | | | |
|  | ⬜ 1 Strongly disagree | | | ⬜ 2 Disagree | ⬜ 3 Somewhat disagree | ⬜ 4 Neutral | ⬜ 5 Somewhat agree | ⬜ 6 Agree | ⬜ 7 Strongly agree |
| **62.** | | I intend to do medical tests **after today** for **early detection/treatment of nose and throat cancer**. | | | | | | | |
|  | ⬜ 1 Strongly disagree | | | ⬜ 2 Disagree | ⬜ 3 Somewhat disagree | ⬜ 4 Neutral | ⬜ 5 Somewhat agree | ⬜ 6 Agree | ⬜ 7 Strongly agree |
